# Histamine Receptors: Ex Vivo Functional Studies Enabling the Discovery of Hits and Pathways

**DOI:** 10.3390/membranes13120897

**Published:** 2023-12-02

**Authors:** Andrea Seldeslachts, Steve Peigneur, Jan Tytgat

**Affiliations:** Toxicology and Pharmacology, KU Leuven, Campus Gasthuisberg, O&N2, Herestraat 49, P.O. Box 922, 3000 Leuven, Belgium; andrea.seldeslachts@kuleuven.be

**Keywords:** histamine receptors, GPCR, *Xenopus laevis* oocytes, two-electrode voltage-clamp, drug discovery

## Abstract

Histamine receptors (HRs) are G-protein-coupled receptors involved in diverse responses triggered by histamine release during inflammation or by encounters with venomous creatures. Four histamine receptors (H1R–H4R) have been cloned and extensively characterized. These receptors are distributed throughout the body and their activation is associated with clinical manifestations such as urticaria (H1R), gastric acid stimulation (H2R), regulation of neurotransmitters in neuronal diseases (H3R), and immune responses (H4R). Despite significant homologous overlap between H3R and H4R, much remains unknown about their precise roles. Even though some drugs have been developed for H1R, H2R, and H3R, not a single H4R antagonist has been approved for clinical use. To enhance our understanding and advance innovative therapeutic targeting of H1R, H2R, H3R, and H4R, we established a robust ex vivo functional platform. This platform features the successful heterologous expression of H1R–H4R in *Xenopus laevis* oocytes, utilizing an electrophysiological readout. Our findings contribute to a deeper understanding of the function and pharmacological properties of the histamine receptors. Researchers can benefit from the utility of this platform when investigating the effects of histamine receptors and exploring potential therapeutic targets. In doing so, it broadens the horizon of drug discovery, offering new perspectives for therapeutic interventions.

## 1. Introduction

**Figure 1:**
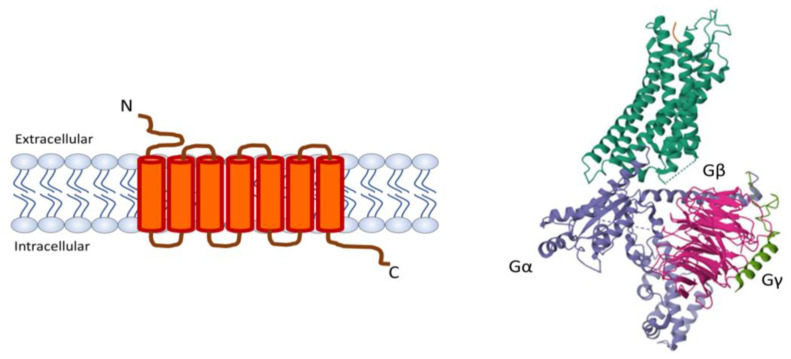
**The structure of G-protein coupled receptors (GPCR)**. (**Left**) Extracellular N-terminus, seven transmembrane helices with three extracellular loops, three intracellular loops, and an intracellular C-terminus. (**Right**) Structure of a GPCR (dark green) in complex with heterotrimeric Gα (purple), Gβ (pink), and Gγ (light green) (PDB: 7L0Q).

G-protein coupled receptors (GPCRs) continue to hold a prominent position among drug targets, with 30% to 40% of existing pharmaceuticals designed to modulate their signaling pathways [1]. In particular, 14% of these compounds offer novel and safe avenues that interact with specific histamine receptors [2]. In the human body, four types of histamine receptors (H1R–H4R) reside. These receptors belong to the aminergic clade of class A (rhodopsin-like) GPCRs and exhibit a shared structural feature, encompassing an extracellular N-terminus, seven transmembrane helices, three extracellular loops, three intracellular loops, and an intracellular C-terminus (Figure 1) [3]. H1R–H4R are widely distributed in the body and have been at the center of attention due to their role in allergic reactions, inflammation, gastric acid regulation, neurotransmission, immune modulation, and envenomation by venomous animals [4,5].

**Figure 2:**
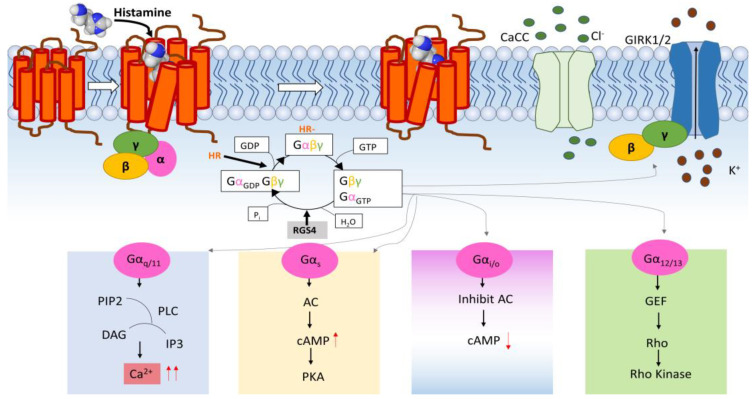
**Histamine receptor—mediated downstream signaling cascades**. Upon GPCR activation by histamine, the heterotrimeric G protein will dissociate into two subunits: Gα and Gβγ. Gα initiates a distinct intracellular signaling cascade: Gα_q/11_, Gα_s_, Gα_i/o_, and Gα_12/13_. Gα_q/11_ stimulates PLC, which will eventually regulate Ca^2+^ signaling and PKC activity. The elevated Ca^2+^ levels also act on the endogenous calcium-activated chloride channels (CaCC). Gα_s_ will activate AC, which will result in an accumulation of intracellular cAMP and activation of PKA. Gαi/o will inactivate AC and thus reduce the cAMP levels. Gα_12/13_ will participate in the regulation of the Rho GTPase signaling pathways (GEF = guanine nucleotide exchange factors). The Gβγ subunit can interact with an effector channel, the G protein-coupled inward rectifying potassium channels (GIRK). The activation of the GIRK channel due to the GPCR signaling will cause a flow of potassium ions (K^+^) out of the cell. RGS4 accelerates the intrinsic GTPase activity of G proteins, facilitating the exchange of GTP for GDP.

As shown in Figure 2, upon histamine binding, a conformational change occurs in a Mg^2+^-dependent manner. Following this shift and interaction, the release of guanosine diphosphate (GDP) from the resting G-protein is triggered, which will stabilize an empty G protein conformation (Gαβγ) [6,7]. Subsequently, the binding of guanosine triphosphate (GTP) affects three conformationally flexible switch regions within Gα, ultimately resulting in the dissociation of the G protein into two distinct subunits (Gα and Gβγ), with GTP bound to the alpha subunit. 

In the human genome, 16 genes encode for Gα including the four subfamilies Gα_q/11_, Gα_s_, Gα_i/o_, and Gα_12/13_, which are pivotal intermediaries in the GPCR signaling yielding different biological outcomes as shown in Figure 2 [8]:

(1) The Gα_q/11_ proteins will activate phospholipase C (PLC), leading to the generation of inositol triphosphate (IP3) and diacylglycerol (DAG) from phosphatidylinositol 4,5-bisphosphate (PIP2). IP3 triggers the release of calcium ions (Ca^2+^) from the intracellular Ca^2+^ stores and DAG will activate protein kinase C (PKC), which will further stimulate various downstream cellular responses to cause, for instance, an allergic reaction.
(2) The Gα_s_ proteins activate the adenylate cyclase (AC) enzyme that will catalyze the conversion of adenosine triphosphate (ATP) to cyclic adenosine monophosphate (cAMP). This elevation of cAMP subsequently triggers protein kinase A, which will phosphorylate proton pumps that are essential in regulating gastric acid secretion in the stomach.
(3) The pertussis toxin-sensitive Gα_i/o_ exerts its influence by inhibiting adenylyl cyclase, thus reducing the levels of cAMP, which initiates a cascade of downstream signaling events.
(4) Gα_12/13_ is involved in the activation of the Rho family GTPases such as RhoA, which can result in changes in the actin cytoskeleton that are important for cell migration, adhesion, and shape.

Within the GPCR signaling, each histamine receptor (HR) subtype exhibits its own specific landscape of diversity [3]. In the past, it was commonly held that each type of HR operated through a straightforward signaling pathway, wherein it would trigger the activation of a single G protein family. Nevertheless, it has now become clear that numerous GPCRs (including histamine receptors) have the capacity to adapt and connect with multiple G protein families, thereby initiating distinct sets of pathways [9]. However, the exact mechanism for switching remains poorly understood, although it can be argued that silencing a certain pathway or overexpressing both the receptor and effector proteins can have an influence [8,10].

Traditionally, H1R is known to be found in smooth muscle cells, endothelial cells, and neurons within the central nervous system. It couples mainly with Gα_q/11_ proteins, which underscores its significance in allergic response and central nervous system effects [11]. Nonetheless, recent studies have revealed that H1R can also interact with Gα_s_ and Gα_i/o_, particularly when both the receptor and the G protein are overexpressed [8,10,12,13,14]_._ Apart from the classical H1R, the pharmaceutical industry has expanded its investigation toward H2R as a drug target for controlling gastric acid secretion in conditions like peptide ulcers. H2R is primarily expressed in gastric mucosa and when activated, it will mainly couple to Gα_s_ proteins, which activates the adenylate cyclase pathway. In certain cellular contexts or when the Gα_s_ pathway is suppressed, H2R can transmit signaling through Gα_q_ and Gα_12/13_, as indicated in references [10,15]. While H1R and H2R have received considerable attention in both research and clinical applications, H3R and H4R remain less explored. H3R is almost exclusively expressed in the nervous system where it plays an important role in several brain disorders [3]. For H4R, there is emerging evidence that it is involved in allergies, inflammation, and autoimmune disorders [3]. Both H3R and H4R are recognized as receptors that signal mainly through Gα_i/o_. However, Seibel-Ehlert et al. (2021) have identified the involvement of Gα_q_ in the signaling pathways mediated by H3R and H4R.

On a parallel note, in addition to the triggering of the Gα pathway, the Gβγ subunit is also activated and exerts its influence on different effector channels including the opening of the G protein-coupled inward rectifying potassium (GIRK) channels via Gα_i/o_ and inhibiting the opening of the Ca^2+^ channels (N- and P/Q-type) [16]. GIRK channels are members of a family of inwardly rectifying potassium (Kir) channels comprising four mammalian subunits: GIRK1 (Kir3.1), GIRK2 (Kir3.2), GIRK3 (Kir3.3), and GIRK4 (Kir3.4) that form homotetramers (GIRK2) and heterotetramers (e.g., GIRK1/GIRK2) [17]. These GIRK channels are essential regulators of controlling cellular excitability and maintaining resting membrane potential. Typically, they become activated by GPCRs. Upon activation, there is a selective outward flux of potassium ions (K^+^) across the cell membrane. This induces membrane hyperpolarization, diminishing the probability of action potential firing and consequently lowering cell excitability.

HRs have been the focus of clinical application for some time and several pharmaceutical companies have developed a variety of medications designed to modulate the H1R, H2R, and H3R activity [16,18]. Nevertheless, despite decades of research and development, the available and commonly used medications are still restricted to three main categories. (1) The first-generation antihistamines such as diphenhydramine (associated with side effects) and the second-generation antihistamines like loratadine (known for reduced side effects). Both are primarily used to alleviate allergic responses mediated by H1R. (2) H2R blockers such as cimetidine and famotidine are employed for managing gastric acid-related disorders. In some cases, these H2R blockers may not provide sufficient relief, prompting a switch to more potent proton-pump inhibitors [3]. (3) Pitolisant, an H3R antagonist, is utilized in the treatment of excessive daytime sleepiness [3,19,20]. Up to now, not a single H4R antagonist has been approved for clinical use, even though some drug candidates reached clinical trials [2,21]. The primary reason is the limited understanding of the pharmacology of H3R and H4R, despite the accumulating evidence and promising interplay in immune-related disorders (H4R) and conditions like Alzheimer’s disease and Parkinson’s disease (H3R) [22,23,24].

The development of medication that selectively and potently blocks the H1–H4 receptors with minimal side effects remains an ongoing challenge. Therefore, researchers need a robust functional bio-assay that serves as an invaluable tool for measuring the interaction between ligands, both agonists and antagonists, and histamine receptors in order to unravel unknown molecular mechanisms. As such, the assay can help to identify novel compounds (hits).

Therefore, in this research, we established and validated an ex vivo functional platform with the successful heterologous expression of the four types of histamine receptors in *Xenopus laevis* oocytes using an electrophysiological readout, enabling the discovery of selective and potent HR-hits and pathways.

For this assay, the most straightforward and quantitative assessment of the histamine receptor function involves the direct electrical recording of ion flow across the cell membrane of *Xenopus laevis* oocytes. It is crucial to note that the measured currents are influenced by the activity of the specific histamine receptor subtype expressed on the cell membrane [25]. The nuanced interplay between K^+^, Ca^2+^, and Cl^−^ currents add a layer of complexity to our observations, necessitating a detailed exploration of the individual contributor to the recorded current. In the context of histamine receptor signaling under normal physiological conditions, calcium ions are primarily associated with the activation of H1R where the G_q/11_ pathway is initiated. The elevation of intracellular calcium levels acts as a signaling amplifier, orchestrating downstream events in the cellular response. Two notable calcium channels are involved: IP3 channels (as discussed above) and calcium-activated chloride channels (CaCC). CaCC are responsive to the changes in intracellular calcium concentration, facilitating the flux of chloride ions [26]. The resultant chloride current contributes to the overall electrophysiological response observed during H1R signaling. The regulation of these channels is important for coordinating the events triggered by histamine such as smooth muscle contraction, neurotransmitter release, and other physiological responses [26]. The activation of H2R, H3R, and H4R via the G_i/o_ signaling pathway in a high potassium extracellular environment has an impact on the potassium ion movement via the effector channels, GIRK. More specifically, the opening of GIRK channels by the activation of H2R, H3R, and H4R allows for an influx of potassium ions into the cell, leading to depolarization of the cell membrane [17]. This potassium current contributes to the overall electrophysiological response observed for H2R, H3R, and H4R.

The resultant chloride current for H1R and potassium current for H2R, H3R, and H4R can be measured using electrophysiological techniques such as a two-electrode voltage clamp, where the membrane potential is controlled while the resulting current is recorded. The change in current is equal in amplitude but opposite in sign (depending on the charge of the ions). This implies that when anions enter (or cations exit), it represents an outward or positive current and the influx of cation (efflux of anions) as an inward or negative current.

## 2. Materials and Methods

### 2.1. Xenopus Laevis Frogs

In this research, all *Xenopus laevis* frog experiments and procedures were approved by the Ethical Committee for Animal Research of KU Leuven (Project No. P186/2019 and No. P074/2023) and were in agreement with the guidelines of the European Union (EU) concerning the welfare of laboratory animals, as declared in Directive 210/63/EU.

### 2.2. Isolation of Xenopus laevis Oocytes by Partial Ovariectomy

Prior to the harvesting of stage V–VI oocytes from ovarian tissue, adult female *Xenopus laevis* frogs were immersed in an aqueous solution containing 0.1% buffered tricaine (ethyl 3-aminobenzoate methanesulfonate, 1 g/L, Sigma-Aldrich, Rockville, MD, USA) and NaHCO_3_ (sodium bicarbonate, 1 g/L; Sigma-Aldrich, Rockville, MD, USA) in aquarium water (pH 7.5) for 15 min. After the recovery period, frogs were monitored daily and placed back in their tanks at the Aquatic Facility of KU Leuven.

The surgically removed ovarian lobes were enzymatically defolliculated in a Ca^2+^-free ND96 solution (96 mM NaCl (Merck, Darmstadt, Germany), 2 mM KCl (AppliChem GmbH, Germany), 2 mM MgCl_2_ (Merck, Darmstadt, Germany), and 5 mM HEPES (Acros Organics, Geel, Belgium) supplemented with collagenase from Clostridium histolyticum type IA (1.5 mg/mL; Sigma-Aldrich, Rockville, MD, USA) on a rocker platform at 16 °C for 2 h and 30 min. After enzymatic defolliculation, the oocytes were transferred to a calcium-containing ND96 buffer (96 mM NaCl, 2 mM MgCl_2_, 2 mM KCl, 5 mM HEPES, and 1.8 mM CaCl_2_ with a pH of 7.5) supplemented with geomycine (100 mg/L; Schering-Plough, Heist-op-den-Berg, Belgium) and theophylline (90 mg/L; ABC chemicals, Nazareth, Belgium) at 16 °C.

### 2.3. In Vitro Synthesis of Messenger RNA

For heterologous expression of histamine receptors in *Xenopus Laevis* oocytes, human H1–H4 receptor cDNA clones in the pcDNA3.1(+) vector were purchased from the cDNA Resource Center (Bloomsburg University Foundation, Bloomsburg, PA, USA). Other plasmids used in this study were obtained from various sources. The plasmid mGIRK1-pSPOR was provided by Kazutaka Ikeda (The Institute of Physical and Chemical Research, RIKEN, Hirosawa, Wako, Japan). We received mGIRK2-pBScMXT as a gift from Henry Lester (California Institute of Technology, Pasadena, CA, USA), and mIRK1 was granted by Lily Jan (Howard Hughes Medical Institute, Chevy Chase, MD, USA). Additionally, hRGS4-pGEM-HE was previously constructed in our lab [27]. To express the corresponding cDNA, we transformed competent *E. coli* JM109 cells (Promega, Madison, WI, USA). Each plasmid for the different channels or receptors underwent linearization with specific enzymes: XhoI for hH1R, hH2R, and hH4R; XbaI for hH3R; EcoRI for mGIRK1; SalI for mGIRK2; NheI for hRGS4-pGEM-HE. Next, cRNA was transcribed with a T7 mMESSAGEmMACHINE transcription kit (Ambion, Austin, TX, USA) for H1R–H4R and hRGS4, SP6 for mGIRK1, and T3 for mGIRK2. The synthesis of mIRK1 cRNA was previously performed in our lab. The quality of the cRNA and concentration was checked with a spectrophotometer at an absorbance of 260 nm and 280 nm (NanoDrop ND-1000 UV/Vis, Wilmington, DE, USA). Oocytes were injected with 10–50 nL of cRNA at a concentration of 1 ng/nL using a micro-injector (Nanoliter Injector A203XVZ, World Precision Instruments, Sarasota, FL, USA). After injection, oocytes were kept at 16 °C in ND96 buffer supplemented geomycine (100 mg/L; Schering-Plough, Heist-op-den-Berg, Belgium) and theophylline (90 mg/L; ABC chemicals, Nazareth, Belgium). Electrophysiological experiments were conducted after 2–3 days for the expression of GIRK1/2 channels or IRK1 and 4–5 days for the expression of the GPCR-GIRK1/2-RGS4 coupling system.

### 2.4. Electrophysiological Recordings with a Two-Electrode Voltage-Clamp

A two-electrode voltage-clamp (TEVC) GeneClamp 500 amplifier (Molecular Devices, Downingtown, PA, USA) was controlled by a pClamp data acquisition system (Axon Instruments, Union City, CA, USA) and pClamp Clampex 10.4 software (Axon Instruments^®^, Scottsdale, AZ, USA), was used to measure the currents across the cell membrane. The whole-cell currents from the oocytes were recorded at room temperature (18–22 °C). Two micro-electrodes, voltage and current electrodes, were fabricated from borosilicate glass capillaries (1.14 mm outside diameter, 0.7 mm inside diameter), pulled by a microelectrode puller, PUL-1 (World Precision Instruments, Sarasota, FL, USA), filled with 3 M KCl by using a MicroFil needle and with a resistance maintained between 0.5 MΩ and 1.5 MΩ. During the measurements, oocytes were placed in a 200 µL recording chamber and continuously perfused with ND96 solution controlled by a perfusion system with gravity flow at 1 mL/min.

For GIRK1/2 and IRK1 measurements, oocytes were voltage-clamped at −90 mV and currents were measured by exchanging a low potassium ND96 solution with a high-potassium solution (HK; 96 mM KCl, 2 mM NaCl, 1 mM MgCl_2_, 1.8 mM CaCl_2_, 5 mM HEPES with a final pH of 7.5). Currents were filtered at 20 Hz and sampled at 100 Hz.

The receptors H2, H3, and H4 were coupled to the inward rectifier potassium channels (GIRK1 and GIRK2) and RGS4 via Gα_i/o_. The first increase in inward K^+^ currents was induced by the HK solution and represents a ‘basal’ K^+^ current (I_K, basal_), indicating receptor-independent GIRK channel activation. In the presence of an agonist, 1 µM histamine in HK, this I_K, basal_ was immediately enhanced and represents the I_K, histamine_. I_K, histamine_ is reversible after histamine washout with HK. This histamine washout period with HK was recorded. Oocytes were voltage-clamped at −90 mV and currents were sampled at 100 Hz and filtered at 20 Hz.

The H1 receptor couples with Gα_q/11_ proteins that are responsible for the release of Ca^2+^ from the intracellular stores. This rise in intracellular Ca^2+^ activates the Ca^2+^-activated Cl^−^ channels that are used as sensitive readout. The currents evoked by 1 µM histamine were sampled at 1000 Hz, filtered at 20 Hz, and measured using a 2-s voltage ramp protocol applied from −120 to +70 mV from a holding potential of −20 mV during perfusion.

The dose–response curve of H1R, H2R, H3R, and H4R was made by exposing oocytes to an increasing histamine concentration until the maximum response was observed. The histamine-evoked currents were measured at each concentration and normalized against the saturated histamine-evoked current.

### 2.5. Chemicals

Several concentrations of histamine (Sigma-Aldrich, Rockville, MD, USA) (agonist) were used for the measurements and to make a dose–response curve for H1R, H2R, H3R, and H4R. In the experiments with pertussis toxin (PTX) (Sigma-Aldrich, Rockville, MD, USA), oocytes were incubated in ND96 solution containing 2.5 ng PTX for 16 h before measurements in order to check the blocking effect on the GPCR signaling. In niflumic acid experiments, H1R injected oocytes were continuously perfused with ND96 solution containing 100 µM niflumic acid (Sigma-Aldrich, Rockville, MD, USA), followed by ND96 solution containing 100 µM niflumic acid together with 1 µM histamine. Niflumic acid is a potent and reversible blocker of the calcium-activated chloride channels (CaCC). A total of 24 nM JNJ 7777120 (Sigma-Aldrich, Rockville, MD, USA) was used as a potent H4R antagonist.

### 2.6. Data and Statistical Analysis

Electrophysiological data were obtained using pClamp Clampex 10.4 (Axon Instruments, San Jose, CA, USA), analyzed using pClamp Clampfit 10.4 (Axon Instruments, San Jose, CA, USA), and presented as the means ± standard error of the mean (SEM) of *n* ≥ 3 independent experiments unless otherwise indicated.

Dose–response curves were generated to assess the relationship between the concentration of histamine and the biological response of the histamine receptor subtypes using Origin 9.0 software (Origin Lab, Northampton, MA, USA). The four-parameter Hill equation was considered appropriate for data fitting due to its ability to capture sigmoidal relationships commonly observed in dose–response curves. The Hill coefficient was allowed to vary, providing flexibility to capture potential variation in cooperativity or interaction. The Equation (1) used is given by:

(1) $$y=A1+\frac{A2-A1}{1+10^{\left(Logx0-x\right)p}}$$

- *A1* bottom asymptote;
- *A2* top asymptote;
- *Logx0* center;
- *P* hill slope.

Statistical analyses were performed to evaluate the goodness-of-fit of the dose–response curves. The reduced chi-squared and adjusted R-squared were used as performance criteria for model selection. For the reduced chi-squared, a good fit value is typically close to 1. Values between 0 and 1 indicate a well-fitted model, illustrating that the model adequately represents the observed data. A value for the adjusted R-squared closer to 1 represents a good fit value. The calculated EC_50_ is presented as the means ± standard deviation (SD) of *n* ≥ 3. The dose–response curves were plotted as a percentage of the activation of histamine against the logarithm of the different concentrations tested. By transforming the concentrations, we aimed to stabilize the variance across the range of concentrations, ensuring a more consistent spread of the biological responses to meet the homogeneity of variance. All experiments were repeated at least three times (*n* ≥ 3).

GraphPad Prism (version 8.1.2, GraphPad Software, San Diego, CA, USA) was employed for statistical analysis of the 1 µM histamine washout period for H2R, H3R, and H4R. Normality was tested before running the Kruskal–Wallis test. The graph represents the mean values ± SD. Significance was determined at *p* ≤ 0.05. Statistical significance levels are denoted as follows: * *p* < 0.05, ** *p* < 0.01, *** *p* < 0.001, **** *p* < 0.0001. Washout periods were recorded for at least three independent cells per histamine receptor subtype (H2R, H3R, and H4R) (*n* ≥ 3).

## 3. Results

To establish and validate an ex vivo functional platform with the heterologous expression of the four histamine receptors, we used an electrophysiological readout system. Due to the distinct readouts associated with the four histamine receptors in our platform, we have opted to describe them in separate groups. H1R was measured using chloride current as the readout, while H2R, H3R, and H4R were assessed with the potassium current as the readout. This differentiation in readouts allowed us to tailor our approach to each histamine receptor group, optimizing the precision of our measurements and enhancing the understanding of their individual signaling pathways.

### 3.1. Functional Expression of Histamine 1 Receptor (H1R) in Xenopus laevis Oocytes and Validation of Signaling via Gαq/11 Proteins

**Figure 3:**
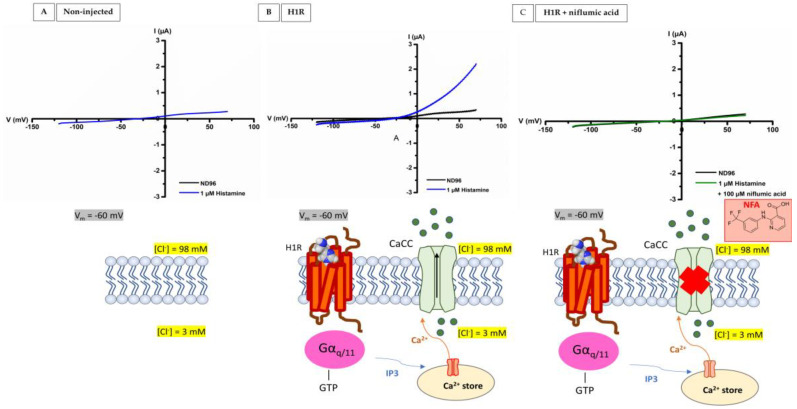
**Activation of histamine 1 receptor (H1R) by histamine**. (**A**) No effect of 1 µM histamine on non-injected oocytes. (**B**) A representative current trace of H1R-injected oocytes was measured with a ramp protocol from −120 to +70 mV from a holding potential of −20 mV. Upon 1 µM histamine application (blue), a shift in the reversal potential (E_rev_) from −50 mV to −20 mV was visible. (**C**) A representative current trace of H1R injected oocytes treated with 100 µM niflumic acid. No appreciable current enhancement can be seen by 1 µM histamine for oocytes treated with 100 µM niflumic acid. All experiments were repeated at least three times (*n* ≥ 3).

We have established and validated a reliable ex vivo functional platform with the successful heterologous expression of the human H1R in *Xenopus laevis* oocytes. First, a ramp protocol (−120 mV to +70 mV) was employed to assess the impact of 1 µM histamine on non-injected oocytes. This initial testing aimed to demonstrate that histamine does not exert a substantial effect on the endogenous ion channels and receptors present in oocytes. As illustrated in Figure 3A, the data clearly indicate that histamine (depicted in blue) lacks a significant impact on non-injected oocytes. Subsequently, we injected oocytes with human H1R RNA and evaluated the influence of 1 µM histamine in the bio-assay. The results indicate that the application of 1 µM histamine led to a shift in the reversal potential (E_rev_) from −50 mV to −20 mV (Figure 3B). This observation aligns with the notion that upon H1R activation, the Gα_q/11_ pathway is triggered, which will release Ca^2+^ from the intracellular calcium stores and activate Ca^2+^-activated Cl^−^ channels (CaCC). Depending on the holding potential, an inward or outward current can be observed. For example, at a potential of −60 mV, the electrochemical gradient favors the movement of chloride ions out of the cell. The movement of anions out of the cell represents a small inward current (blue). At a potential of +60 mV, the electrochemical gradient favors the movement of chloride ions in the cell. This movement represents a big outward current (blue). To prove the presence of Gα_q/11_ signaling in the oocytes, H1R-injected oocytes were treated with 100 µM niflumic acid. Niflumic acid is a potent reversible blocker of the sensitive Ca^2+^ activated Cl^−^ channel currents. This means that when Ca^2+^-activated Cl^−^ channels are blocked, an increase in Ca^2+^ levels due to the activation of H1R by histamine and initiation of the G_q/11_ pathway will not cause a chloride current as readout. This mechanism is clearly visible in Figure 3C: 1 µM histamine + 100 µM niflumic acid (green) was not able to cause a significant change in conductance. Overall, we provide evidence that 1 µM histamine does not stimulate the activity of endogenous ion channels and receptors in non-injected oocytes. Moreover, the experiments establish that the enhanced outward results from the stimulation of Ca^2+^ activated Cl^−^ channels through the G_q/11_ signaling pathway.

### 3.2. Successful and Robust Heterologous Expression Platform to Measure H2R, H3R, and H4R Coupled with GIRK1/2 and RGS4 in Xenopus laevis Oocytes

**Figure 4:**
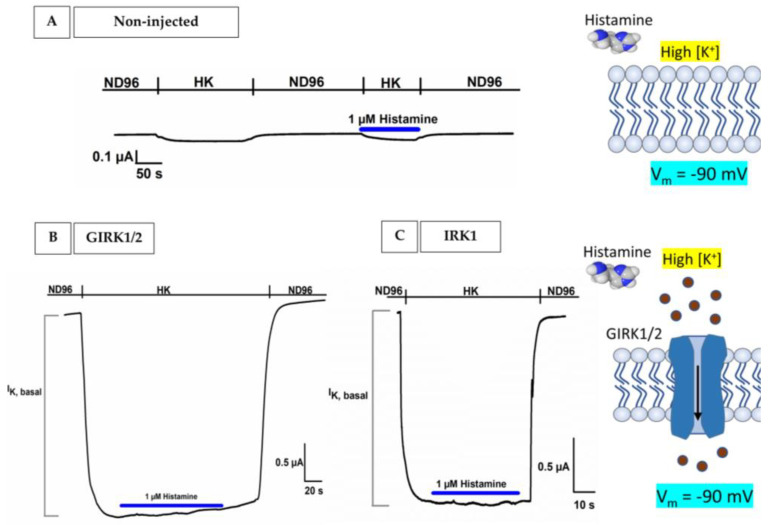
**Evaluation of the observations and function of the bio-assay**. The current is visible on the *y*-axis, the time is visible on the *x*-axis, and the holding potential is −90 mV. (**A**) A representative current trace showed no significant currents induced by the application of HK and 1 µM histamine + HK (blue) in the non-injected oocytes. (**B**,**C**) A representative current trace shows that K^+^ currents were induced by the HK solution and represents a ‘basal’ K^+^ current (IK, basal), indicating a receptor-independent GIRK channel activity. No significant currents were induced by the application of 1 µM histamine + HK (blue) in the GIRK1/2 injected oocytes and IRK1 injected oocytes.

For the expression of the human H2R, H3R, and H4R, we used the availability of the intrinsic pertussis toxin (PTX) sensitive Gα_i/o_ to couple with the inward rectifier potassium channels GIRK1/2 and RGS4 in oocytes. To evaluate and confirm the functional properties of the bio-assay, some control experiments were performed following the pathway. First, non-injected oocytes were tested for sensitivity toward a high potassium concentration solution (96 mM KCl, referred to as HK) and histamine dissolved in HK. A negligible intrinsic current (I_K, intrinsic_) can be seen in Figure 4A and presumably originates from endogenous inward rectifier channels. Changing from HK to 1 µM histamine + HK did not yield any significant change in membrane current, illustrating that histamine does not act on endogenous receptors and/or ion channels in non-injected oocytes.

Next, the effect of HK + 1 µM histamine was investigated on oocytes injected with only GIRK1/2 and IRK channels. Under normal physiological conditions (2 mM KCl in ND96 solution) and a holding potential set at −90 mV, there was no noticeable inward or outward current in the ND96 solution since this corresponded to the equilibrium potential for potassium ions (K^+^), as shown in Figure 4B,C. Upon replacing the ND96 solution with a solution featuring a high potassium concentration (96 mM KCl, HK), there will be a shift to the right in the current–voltage relationship to a new E_K_ (depolarization of the membrane potential). As a result, at a holding potential of −90 mV, a substantial inward current I_K, basal_ can be seen in Figure 4B,C. Here, the increase in inward K^+^ currents was induced by the HK solution and represents a ‘basal’ K^+^ current (I_K, basal_), indicating receptor-independent GIRK channel (Figure 4B) and IRK channel (Figure 4C) activation. No significant currents were induced by the application of 1 µM histamine + HK (blue) in GIRK1/2 injected oocytes and IRK1 injected oocytes.

**Figure 5:**
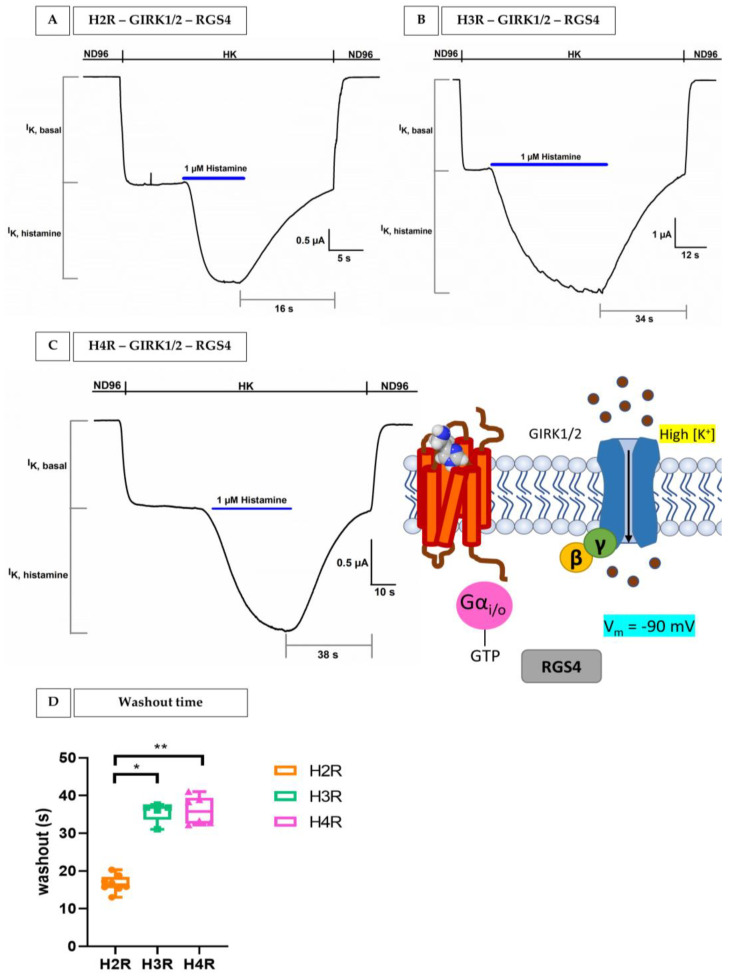
**The activation of H2R, H3R, and H4R by histamine in the H2R/H3R/H4R–GIRK1/2-RGS4 coupling system expressed in oocytes**. The current is visible on the *y*-axis and the time is visible on the *x*-axis. (**A**) Oocytes co-injected with cRNAs of H2R, GIRK1/2 channels, and RGS4 proteins. (**B**) Oocytes co-injected with cRNAs of H3R, GIRK1/2 channels and RGS4 proteins. (**C**) Oocytes co-injected with cRNAs of H4R, GIRK1/2 channels, and RGS4 proteins. I_K, basal_ was observed by exchanging the normal physiological solution (ND96, 2 mM KCl) for a solution containing a high potassium concentration (HK, 96 mM). I_K, histamine_ was observed by exchanging HK to 1 µM histamine (blue) + HK. (**D**) Time to washout of 1 µM histamine of H2R, H3R, and H4R in the H2R/H3R/H4R-GIRK1/2–RGS4 coupling system expressed in oocytes. The graph represents the washout time in seconds versus the specific histamine receptor subtype. Differences were considered significant if the *p* value was smaller or equal to 0.05. For the difference between H2R and H3R, the * *p* value was 0.017 and for the difference between H2R and H4R, the ** *p* value was 0.0030. All cells were voltage-clamped at −90 mV, and experiments were repeated at least three times (*n* ≥ 3).

After these two control experiments, we coupled the histamine receptor subtypes (H2R, H3R, and H4R) with the GIRK1/2 channels and RGS4 in oocytes. This created a robust and quantifiable signal for H2R in Figure 5A, H3R in Figure 5B, and H4R in Figure 5C, measured at a holding potential of −90 mV. Here, the first increase in inward K^+^ currents was induced by the HK solution and represents a ‘basal’ K^+^ current (I_K, basal_), indicating receptor-independent GIRK channel activation. Next, in the same figures, an enhancement in I_K, basal_ can be seen (I_K, histamine_). This visible enhancement, I_K, histamine_, was mediated by the addition of an agonist, 1 µM histamine in HK. The binding of histamine to H2R, H3R, or H4R promoted the activation and thus dissociation of G protein in two subunits, Gα_i/o_ and Gβγ protein. The Gα_i/o_ subunit reduces the cAMP levels and the Gβγ subunit will bind and activate the opening of the GIRK1/2 channel, which causes an influx of K^+^ in the cell (I_K, histamine_). I_K, histamine_ is reversible after a washout period with HK.

Important to note the differences in terms of the washout period between H2R (Figure 5A, 16 s), H3R (Figure 5B, 35 s), and H4R (Figure 5C, 38 s) in the representative current traces. The average 1 µM histamine washout period for H2R, H3R, and H4R is shown in Figure 5D. Here, it can be seen that 1 µM histamine will remain bound to H3R and H4R for a significantly longer time compared to H2R.

**Figure 6:**
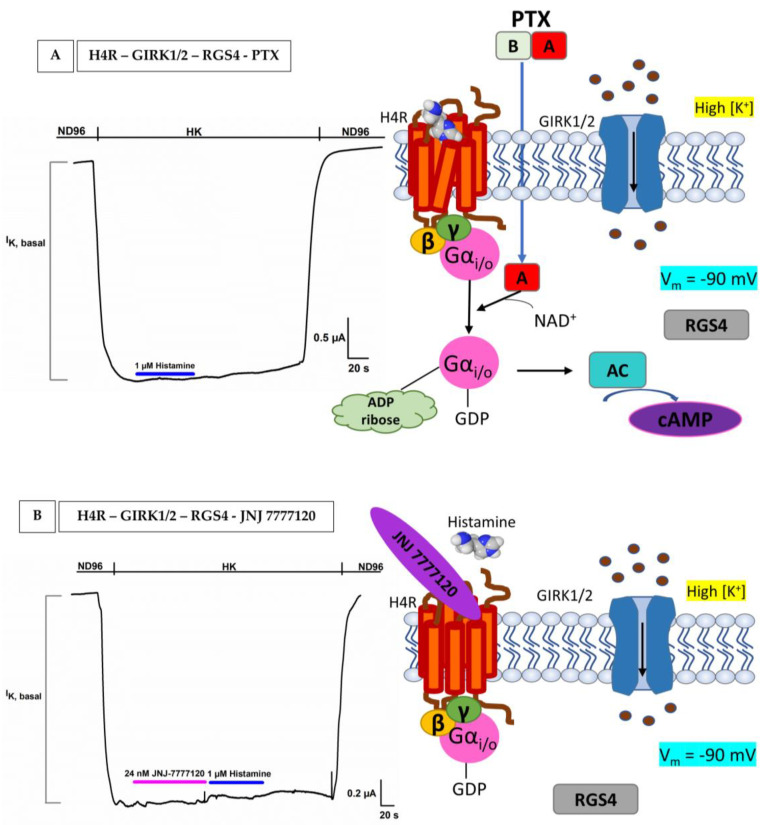
**The effect of PTX and a specific H4R antagonist on H4R injected oocytes**. The current is visible on the *y*-axis and the time is visible on the *x*-axis. (**A**) No alteration of the current was observed after the addition of 1 µM histamine (blue) to H4R co-injected GIRK1/2–RGS4 injected oocytes after PTX treatment. Upon penetration of the A-protomer of PTX in the oocytes, ADP-ribosylation occurred at the cysteine residue of Gα_i/o_, leading to the inactivation of Gα_i/o_. Consequently, the suppressive impact of Gα_i/o_ on adenylate cyclase (AC) activity diminished, causing an elevation in intracellular cAMP levels. (**B**) A representative trace of H4R-GIRK1/2–RGS4 showed that the effect of 1 µM histamine (blue) was blocked by JNJ7777120 (pink). All cells were voltage-clamped at −90 mV and experiments were repeated at least three times (*n* ≥ 3).

To provide additional confirmation of the GPCR–GIRK linkage and ascertain the presence of histamine receptors, we incorporated two supplementary control experiments. In Figure 6A, oocytes were co-injected with H4R-GIRK1/2–RGS4 and the pertussis toxin. In the absence of PTX, Gα_i/o_ leads to an inhibition of adenylate cyclase, resulting in a decrease in cAMP levels. However, in the presence of PTX, there is an uncoupling of the system. This uncoupling allows adenylate cyclase to maintain its activity, leading to the (re)formation of cAMP. It is important to note that the uncoupling, which results in increased cAMP, is not measured in our system. Instead, what we measured is the fact that the uncoupling no longer produces GαGTP, thereby preventing the activation of GIRK1/2. The latter is clearly evident in Figure 6A, where 1 µM histamine failed to increase I_K, basal_, resulting in the absence of I_K, histamine_.

In the next validation experiment, as shown in Figure 6B, we assessed the impact of histamine on oocytes co-injected with H4R and GIRK1/2 in the presence of a potent and sticky antagonist of H4R, 24 nM JNJ 7777120 with an IC_50_ of 5 ± 0.1 nM [28]. The application of 24 nM JNJ 7777120 was able to effectively inhibit all H4Rs signaling within the same cell. Due to the prior blockade of H4Rs by the antagonist, there was no observable current increase by histamine or I_K, histamine_.

From these results, we can conclude that we built a successful and robust heterologous expression platform to measure H2R, H3R, and H4R coupled with GIRK1/2 and RGS4 in *Xenopus laevis* oocytes.

### 3.3. Concentration–Response Relationship of the Effects of Histamine on H1R and on H2R/H3R/H4R-GIRK1/GIRK2-RGS4 Coupling System Expressed in Xenopus laevis Oocytes

**Figure 7:**
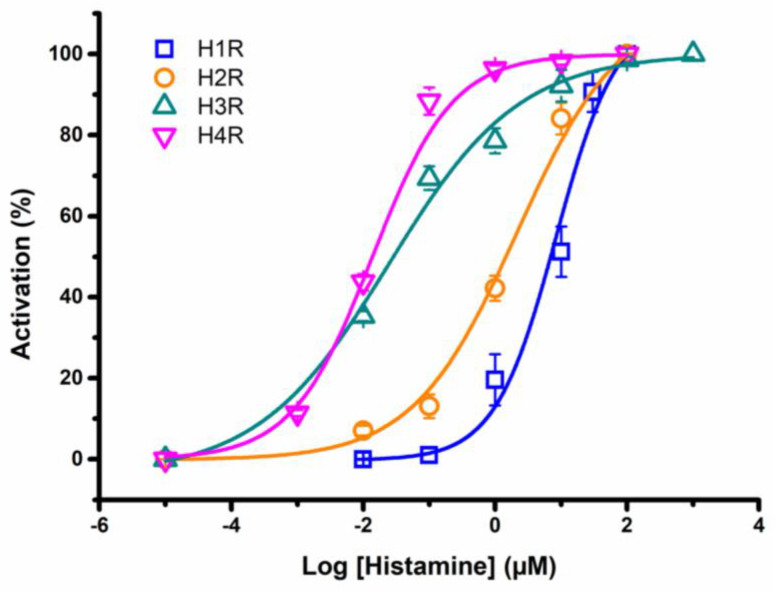
**Activation–response curve of H1R, H2R, H3R, and H4R**. The percentage of activation of histamine in H1R (blue), H2R (orange), H3R (green), or H4R (pink) was plotted against the logarithm of the different concentrations tested. The corresponding EC_50_ value for H1R, H2R, H3R, and H4R yielded 8.4 ± 3.1 µM, 2.1 ± 1.1 μM, 0.024 ± 0.0012 µM, and 0.013 ± 0.0011 µM histamine, respectively. The visualized error bars represent the standard error of the mean (S.E.M). All experiments were repeated at least three times (*n* ≥ 3).

After having established the activation of the H1R and H2R, H3R and H4R coupling with the GIRK1/2 and RGS4 system and to further validate the bio-assay, we constructed an activation response curve. In this study, oocytes were subjected to a rising series of histamine concentrations and the elicited currents were normalized against the saturated histamine concentration. The currents were measured via a voltage-ramp protocol for H1R with a holding potential of −90 mV for H2R, H3R, and H4R. The dose–response curve is presented with the percentage of activation plotted against the logarithmically scaled concentrations in Figure 7.

As depicted in blue, the concentration–response curve for H1R. An EC_50_ value of 8.4 ± 3.1 µM was determined, which aligns with the value reported in a previous study (EC_50_ = 24 µM) [29]. For H2R (orange), we observed the maximum current activation at an approximately 100 µM concentration, and the calculated EC_50_ value was estimated at 2.1 ± 1.1 µM. This closely corresponds to the literature value of EC_50_ = 10 µM [30]. For H3R (shown in green), the half-maximum response was achieved at a concentration of 0.024 ± 0.0012 µM, aligning perfectly with the literature-reported EC_50_ = 55 ± 7.89 nM [31]. The determined EC_50_ value for H4R (pink) was 0.013 ± 0.0011 µM, which also closely matches the previously described value of EC_50_ = 13 ± 0.2 nM [28]. Notably, differences in potency were clearly visible in the dose–response curve, confirming that histamine exhibits significantly higher affinity for H3R and H4R, consistent with existing literature.

## 4. Discussion

The results of this study demonstrate the successful establishment and validation of an ex vivo functional platform for investigating the histamine receptor, particularly focusing on human H1R, H2R, H3R, and H4R, in *Xenopus laevis* oocytes. This platform offers valuable insights into the signaling pathways and pharmacological properties of these receptors and opens a unique window to functionally characterize newly developed pharmacologically active compounds on histamine receptors in the quest for novel therapeutics.

In this quest, researchers often use human cells to express histamine receptors. From an experimental point of view, this can be very challenging, mainly because of the receptor complexity, in achieving consistent and sufficient expression levels, and assessing the effects of the downstream signaling pathway and ligand specificity: ligands need to be highly selective to avoid off-target effects [16]. The oocytes from the model organism *Xenopus laevis* used in our platform provide a solution. To begin, the oocyte constitutes an excellent protein factory that is highly efficient in translating and correctly expressing the exogenous micro-injected RNA encoding the four types of human histamine receptors in the cell membrane [32]. Once the histamine receptor was correctly expressed, usually after 48–72 h, a two-electrode voltage-clamp (TEVC) method was used to test their functionality. The TEVC measures the time-dependent change in electrogenic ion fluxes across the membrane: equal in amplitude but opposite in sign (depending on the charge of the ions), which means that when anions enter (or cations exit), it represents an outward or positive current and the influx of cations (efflux of anions) as an inward or negative current [32].

An advantage of our system is that we can study all human HRs side-by-side and that we can overexpress them in high density, which evokes currents in the µA range. As such, these currents are much larger than the background signals induced by endogenous background channels and receptors [33]. These endogenous ion channels/receptors have been extensively studied in oocytes and are closely related to those found in mammalian cell lines such as human embryonic kidney cells (HEK) [34]. In the case of H1R, their presence is actually used as a positive control to indicate the downstream Gα_q/11_ signaling [33,35,36,37]. Oocytes have a powerful intracellular signaling cascade that allows us to measure heterologous expressed human H1R monitored by recording the Ca^2+^-dependent Cl^−^ currents. Our results show that the application of 1 µM histamine induces currents in µA and alters the reversal potential, shifting from the reversal potential of −50 mV to a more depolarized potential of −20 mV. This response aligns perfectly with the activation of the Gα_q/11_ signaling pathway, a well-known downstream cascade of H1R activation. More specifically, in the Gα_q/11_ signaling pathway, DAG/IP3 is formed by the activation of PLC, which will release intracellular calcium ions, in turn activating Ca^2+^-dependent Cl^−^ channels. To strengthen these observations, two control experiments with non-injected oocytes and niflumic acid were performed. Niflumic acid is known to potently but reversibly block Ca^2+^-activated Cl^−^ channels by physically obstructing the ion-conducting pore [38]. In both experiments, we could not observe any significant changes, which provides compelling evidence that this response is indeed a result of the H1R stimulation with histamine followed by the Gα_q/11_ signaling, and shows a robust and consistent signal compared to the low expression of H1R in HL-60 cells [16,39,40].

The discovery of K^+^ channels sensitive to activation by G-proteins in the *Xenopus* system by Kubo et al. and Dascal et al. in 1993 helped to expand the capabilities of our platform to encompass H2R, H3R, and H4R [41]. In our bio-assay, these human H2R, H3R, and H4R can be coupled to co-expressed G protein-coupled inward rectifying potassium (GIRK) channels through endogenous oocyte heterotrimeric G-proteins consisting of Gα_i/o_ and Gβγ and with RGS4. To the best of our knowledge, to date, no evidence has been provided that H2R is capable of activating Gα_i/o_ proteins. The observed connection in our bio-assay might potentially be rationalized by the adjustment of H2R signaling to the overexpression of the G protein effector channel, GIRK1/2, which serves as a readout [9]. This finding demonstrates again that under certain conditions, histamine receptors can adapt and couple to multiple G-protein-mediated signal pathways [8]. Despite this, the exact mechanism behind this adaptation behavior is not known yet [8,9,10].

The fact that we can co-inject different subunits, have the capability of correctly simultaneously expressing them, and thus also investigate the role of each subunit separately are great advantages of the oocyte system over mammalian cell lines with cDNA transfection. Additionally, the level of injection into the cell can be fully controlled and is easy thanks to their big diameter (largest single cells in the animal kingdom) [35]. Nevertheless, one downside of the oocyte system (but also for *E. coli* and *yeast*) is that although oocytes are able to make many post-translational modifications (glycosylation, phosphorylation, acetylation, or folding), they cannot replicate the post-translational modifications performed by the cells that originally expressed them. Mammalian expression systems including HEK293 cells, CHO cells, NIH-3T3 cells, and COS-7 cells are superior for this purpose [16,34,42].

The coupling of H2R, H3R, and H4R with GIRK1/2 and RGS4 allowed us to measure the activity of these receptor subtypes accurately. A −90 mV holding potential protocol revealed successful coupling where a basal K^+^ current (I_K, basal_) was triggered by elevated extracellular K^+^ concentrations, followed by a noticeable increase in this current (I_K, histamine_) upon the addition of 1 µM histamine. This I_K, histamine_ represents the binding of histamine to H2R, H3R, or H4R, followed by the dissociation of the G protein: Gαi/o subunit reduces the cAMP levels and the Gβγ subunit will bind and activate the opening of the GIRK1/2 channel, which causes an influx of K^+^ in the cell (I_K, histamine_), since we are working in a high potassium extracellular concentration and not under physiological conditions. Crucially, several control experiments provided compelling evidence of the functional properties of the bio-assay and shed light on the mechanism and response to histamine receptor activation. In our first control experiment, non-injected oocytes were tested for sensitivity to HK and histamine in HK. For HK, a negligible intrinsic current was visible, which likely arises from endogenous IRK channels. Histamine did not produce any significant change and thus does not act on endogenous receptors or ion channels. Next, we repeated the same experiment, but this time, the oocytes were injected with human IRK1 and GIRK1/2 channels. It is worth noting that IRK1 channels do not interact with G-proteins, which sets them apart from GIRK channels. Identity wise, they are quite similar: 42% between IRK1 and GIRK1 and 45% between IRK1 and GIRK2 [43]. Intriguingly, for both oocytes injected with GIRK1/2 and IRK1, the application of histamine did not evoke a significant change in I_K, histamine_. Moving on to the third control experiment, co-injected oocytes H4R with GIRK1/2 and RGS4 were exposed to pertussis toxin (PTX). In the absence of PTX, the inhibition of adenylate cyclase by GαGTP led to a consequential reduction in cAMP levels. However, the presence of PTX uncoupled this system, allowing adenylate cyclase to persist in its activity, leading to the (re)formation of cAMP. This phenomenon can be explained by the action of the A-protomer of PTX, which deactivates Gαi/o by catalyzing the ADP-ribosylation of a cysteine residue within the α subunit of the heterotrimeric Gα_i/o_ protein [44]. The absence of GαGTP due to uncoupling thereby hinders the activation of GIRK1/2 and this consequently results in the abolishment of I_K, histamine_ when exposed to 1 µM histamine. In the last validation experiment, it was also demonstrated that the action of 1 µM histamine (I_K, histamine_) could be completely blocked by a specific H4R antagonist, JNJ 7777120, using oocytes co-injected with H4R-GIRK1/2 and RGS4. Taking everything together, it is notable that the system nicely mimics the physiological reality of H2R/H3R/H4R coupling with GIRK1/2-RGS4.

The robustness of our system compared to other systems can probably be explained by the presence of the regulator of G protein Signaling 4 (RGS4), which is a modulator of the G-protein deactivation kinetics that will turn off the GPCR signaling more rapidly by facilitating the hydrolysis of GTP (guanosine triphosphate) to GDP (guanosine diphosphate) [45,46,47] (Figure 2). More specifically, RGS4 enhances the activation of GIRK channels, typically by increasing the availability of free Gα_i/o_βγ subunits and not by directly stimulating the GIRK channels. Moreover, there are studies that prove that RGS4 proteins increase the sensitivity for screening potential ligands [47].

To further solidify the reliability of our ex vivo functional platform, we performed dose–response experiments for each histamine receptor subtype. The dose–response curves unveiled distinct affinities for histamine with calculated EC_50_ values of 8.4 ± 3.1 µM for H1R, 2.1 ± 1.1 µM for H2R, 0.024 ± 0.0012 µM for H3R, and 0.013 ± 0.0011 µM for H4R. Remarkably, our EC_50_ values closely mirrored those reported in the previous literature [28,29,30,31]. Furthermore, the differences in affinity were also visible in the washout period. A total of 1 µM histamine bound stronger to H3R and H4R, resulting in a significantly longer washout period compared to H2R. Both observations reinforce the robustness of our experimental system. Importantly, the marked differences in histamine affinities among these receptor subtypes underscore their unique pharmacological properties whereby the imidazole ring interacts with the glutamate residue in transmembrane 5 for H3R and H4R. H3R and H4R show a high degree of sequence similarity and are activated by histamine in the nM range compared to the µM range for H1R and H2R [3].

This result shows that our expression system is well-suited for pharmacological studies and that it can be used to give a complete pharmacological profile of all compounds available/newly developed at all hHRs, for which there is still a need [16]. In this way, we can obtain insights into the potential selectivity, specificity, affinity, and mode of action (agonist, antagonist, inverse agonist) of drugs targeting the histamine receptors and improve target therapy.

## 5. Conclusions

In the road toward novel therapeutics targeting H1R, H2R, H3R, and H4R, we created and confirmed a reliable and powerful ex vivo functional platform using *Xenopus laevis* oocytes and electrophysiological measurements. Beyond elucidating receptor-specific signaling pathways, our system enables researchers to gain insights into the pharmacological properties, concentration–response relationships, and characterize receptor affinity. The findings not only increase the knowledge of histamine receptor function and pharmacology but could be of interest to researchers in drug discovery who are investigating the effect of the histamine receptors (H1R, H2R, H3R or H4R) together with potential therapeutic targets.

## Data Availability

The data presented in this study are available on request from the corresponding author.
